# Myocarditis mimicking acute myocardial infarction

**DOI:** 10.1007/s12471-015-0732-3

**Published:** 2015-07-16

**Authors:** J. van Nierop, A. Limburg, C.E.E. van Ofwegen-Hanekamp

**Affiliations:** 1 Department of Cardiology, Diakonessenhuis, Utrecht, The Netherlands; 2 University Medical Centre Utrecht, Utrecht, The Netherlands

We present a 29-year-old male with fever, acute onset of severe chest pain and short-lasting localised ST elevations on the electrocardiogram (Fig. 1), characteristic for acute myocardial infarction. On echocardiography regional wall motion abnormalities were observed in concordance with the electrocardiogram ST vector. Acute coronary angiography showed normal coronaries. Creatinine kinase-MB and high-sensitive troponin levels were 78 and 1.37 U/l at maximum with a characteristic rise and fall suggesting infarction. We hypothesised coronary artery spasm.

**Fig. 1:**
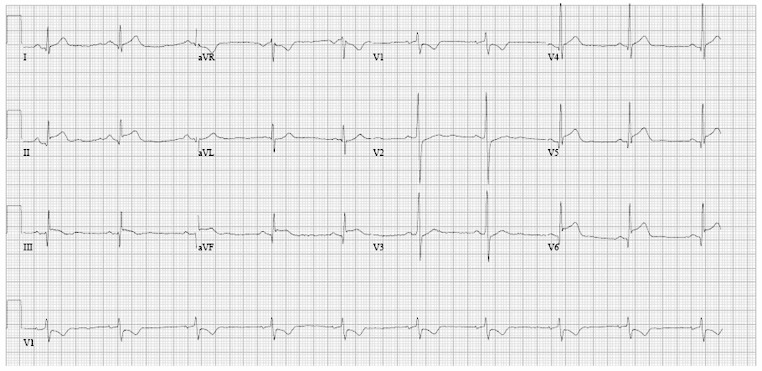
ECG at presentation with inferior and lateral ST elevations and significant anterior ST depressions

Cardiac magnetic resonance (CMR) changed the diagnosis of infarction into myocarditis of the midwall. In retrospect, several clinical features, including leukocytosis and elevated C-reactive protein, could have led to the diagnosis of myocarditis. Lifelong treatment with cardiac medication was prevented. Endomyocardial biopsy is the gold standard for diagnosing myocarditis; however due to its invasive approach it is not common practice [1]. CMR is an accepted diagnostic tool. Image analysis is limited by great clinical variance ranging from chest pain to fulminant heart failure [2, 3]. Using T1 and T2 sequences and late gadolineum enhancement, one can differentiate between myocardial infarction and myocarditis [1]. When Lake-Louise criteria are applied, sensitivity and specificity of CMR proved to be high compared to endomyocardial biopsy [1–4]. Viral myocarditis can induce myocardial ischaemia [1, 4]. The exact pathogenesis is unclear. Hypotheses state that ischaemia is a consequence of local endothelial dysfunction, coronary spasms and in situ thrombi formation [4, 5]. Specific epicardial lesions as are shown in Fig. 2 are proof of myocarditis. If no coronary stenosis is seen on CAG, myocarditis may be the cause.

**Fig. 2:**
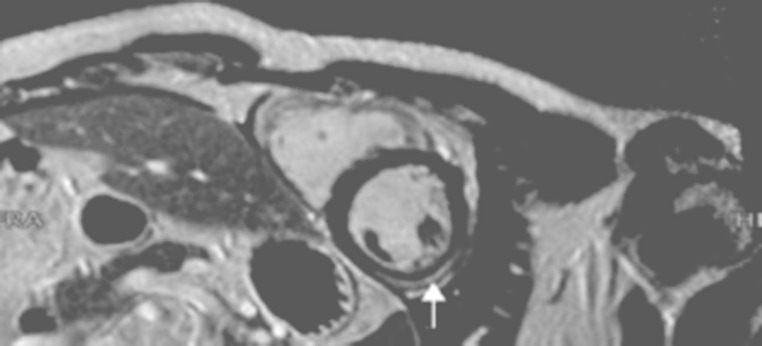
MR image of the patient. The white arrow (gadolinium contrast) points to subepicardial inflammation of the midwall, the clue to the diagnosis of myocarditis

## Conflict of interests

None declared.
